# Correction: Chemical fingerprinting and quantitative monitoring of the doping drugs bambuterol and terbutaline in human urine samples using ATR-FTIR coupled with a PLSR chemometric tool

**DOI:** 10.1039/d0ra90020f

**Published:** 2020-03-04

**Authors:** Faisal K. Algethami, Sherif M. Eid, Khadiga M. Kelani, Mohamed R. Elghobashy, Mohamed K. Abd El-Rahman

**Affiliations:** Chemistry Department, Faculty of Science, Imam Mohammad Ibn Saud Islamic University Riyadh Saudi Arabia; Analytical Chemistry Department, Faculty of Pharmacy, October 6 University 6 October City Giza Egypt Sheriefmohammed@o6u.edu.eg Sherief055@icloud.com +201111609539; Analytical Chemistry Department, Faculty of Pharmacy, Cairo University El-Kasr El-Aini Street ET-11562 Cairo Egypt; Analytical Chemistry Department, Faculty of Pharmacy, Modern University for Technology and Information Cairo Egypt

## Abstract

Correction for ‘Chemical fingerprinting and quantitative monitoring of the doping drugs bambuterol and terbutaline in human urine samples using ATR-FTIR coupled with a PLSR chemometric tool’ by Faisal K. Algethami *et al.*, *RSC Adv.*, 2020, **10**, 7146–7154.

The authors regret that one of the affiliations (affiliation *a*) was incorrectly shown in the original manuscript. The corrected list of affiliations is as shown in this Correction article.

The Royal Society of Chemistry apologises for these errors and any consequent inconvenience to authors and readers.

## Supplementary Material

